# Evaluation of the Biomarkers HMGB1 and IL-6 as Predictors of Mortality in Cirrhotic Patients with Acute Kidney Injury

**DOI:** 10.1155/2020/2867241

**Published:** 2020-09-25

**Authors:** Célio Geraldo de Oliveira Gomes, Marcus Vinicius Melo de Andrade, Ludmila Resende Guedes, Henrique Carvalho Rocha, Roberto Gardone Guimarães, Fernando Antônio Castro Carvalho, Eduardo Garcia Vilela

**Affiliations:** ^1^Postgraduate Programme in Adult Health Applied Sciences, School of Medicine, Federal University of Minas Gerais, Brazil; ^2^Alfa Institute of Gastroenterology, Clinics Hospital, Federal University of Minas Gerais, Brazil

## Abstract

**Background:**

Acute kidney injury (AKI) affects from 20% to 50% of cirrhotic patients, and the one-month mortality rate is 60%. The main cause of AKI is bacterial infection, which worsens circulatory dysfunction through the release of HMGB1 and IL-6.

**Objectives:**

To evaluate HMGB1 and IL-6 as biomarkers of morbidity/mortality.

**Methods:**

Prospective, observational study of 25 hospitalised cirrhotic patients with AKI. Clinical and laboratory data were collected at the time of diagnosis of AKI, including serum HMGB1 and IL-6.

**Results:**

The mean age was 55 years; 70% were male. Infections accounted for 13 cases. The 30-day and three-month mortality rates were 17.4% and 30.4%, respectively. HMGB1 levels were lower in survivors than in nonsurvivors at 30 days (1174.2 pg/mL versus 3338.5 pg/mL, *p* = 0.035), but not at three months (1540 pg/mL versus 2352 pg/mL, *p* = 0.243). Serum IL-6 levels were 43.3 pg/mL versus 153.3 pg/mL (*p* = 0.061) at 30 days and 35.8 pg/mL versus 87.9 pg/mL (*p* = 0.071) at three months, respectively. The area under the ROC curve for HMGB1 was 0.842 and 0.657, and that for IL-6 was 0.803 and 0.743 for discriminating nonsurvivors at 30 days and three months, respectively. In multivariate analysis, no biomarker was independently associated with mortality.

**Conclusion:**

HMGB1 levels were associated with decreased survival in cirrhotics. Larger studies are needed to confirm our results.

## 1. Introduction

Acute kidney injury (AKI) is one of the most serious complications of cirrhosis, affecting from 20% to 50% of hospitalised patients [1, 2]. Approximately 60% progress to death within 90 days [3]. Kidney failure is one of the main defining criteria for acute on chronic liver failure (ACLF), a syndrome that has long been known but has only recently been clearly defined. ACLF is characterised by acute decompensation of liver disease associated with organ failure and high short- and medium-term mortality [4, 5]. Exacerbated systemic inflammation in response to a precipitating factor—bacterial infections and active alcoholism, for example—plays an important role in the development of ACLF [4]. The inflammatory mediators involved in this process include interleukin 6 (IL-6) and High-Mobility Group Box 1 (HMGB1) [4, 6, 7].

HMGB1 is a proinflammatory nuclear protein actively secreted by cells of the innate immune system and released during apoptosis; it is also released by hepatocytes undergoing cell death [8, 9]. HMGB1 is involved in the inflammatory response secondary to drug-induced kidney injury and ischaemia/reperfusion and is also secondary to hepatitis B and C virus infections. It also actively participates in the pathophysiological mechanisms of nonalcoholic fatty liver disease, the progression of hepatic fibrosis, and the regulation of the development of hepatocellular carcinoma [9–11]. When interacting with Toll-like receptor 4 (TLR-4), a type of transmembrane pattern recognition receptor in one of its various signalling pathways, HMGB1 induces the activation of nuclear factor kappa B, producing immunostimulatory responses through transcriptional proinflammatory genes, including tumour necrosis factor, IL-1, and, finally, IL-6 [12]. Similarly, during sepsis, HMGB1 can accumulate in renal tissue and urine, stimulating renal tubular cells, through interaction with TLR-4 receptors, to secrete inflammatory cytokines, including IL-6 [13]. However, the role of these biomarkers as predictors of poor prognosis in cirrhotic patients is not yet known.

This study is aimed at evaluating the role of IL-6 and HMGB1 as predictors of mortality in cirrhotic patients with AKI admitted to the Clinics Hospital of the Federal University of Minas Gerais.

## 2. Methods

This is a prospective, observational study of cirrhotic patients older than 18 years of age hospitalised at the Clinics Hospital of the Federal University of Minas Gerais, diagnosed with AKI at admission or at any time during their hospitalisation. The exclusion criteria were dialysis prior to admission, previous liver or kidney transplant, and cancer (except hepatocellular carcinoma).

AKI was defined using the criteria proposed by the Acute Kidney Injury Network (AKIN) [14] and later revised by the International Club of Ascites (ICA) [15]. The presence of two serum creatinine values with a difference of at least 0.3 mg/dL within 48 hours, or a percentage increase ≥ 50% from the baseline which is known, or presumed, to have occurred within the prior 7 days, was used for the diagnosis of AKI. The baseline creatinine level was defined as the most recent and stable value prior to hospital admission within a maximum period of three months. Response to treatment was defined as complete when there was a return to creatinine value under 0.3 mg/dL of the baseline, incomplete when there was a regression in ICA-AKI stage with reduction of creatinine value of 0.3 mg/dL or more above the baseline value, or absent when there was no regression of AKI, that is, moving into milder ICA-AKI stage.

Patients were classified, from the aetiological point of view, into four groups: infection, hypovolemia, parenchymal renal disease, and hepatorenal syndrome. Once a possible case of AKI was identified, the patient underwent a careful review of their clinical history; full physical examination; laboratory tests including blood count, sodium, potassium, chloride, magnesium, glucose, liver enzymes, albumin, bilirubin, prothrombin time and INR, activated partial thromboplastin time, urinary sodium, urinary creatinine, urinary urea and urinalysis, blood culture, and urine culture; examination of ascitic fluid, if present, through a differential leukocyte count; measurement of lactate dehydrogenase, albumin, and total protein; culture (in blood culture bottles); and ultrasonography of the full abdomen, to establish the aetiology. The management of AKI followed the care protocol used by the Gastroenterology Department of the Clinics Hospital of the Federal University of Minas Gerais: [1] medications that could be associated with AKI are suspended, especially diuretics; [2] volume expansion according to the cause and degree of kidney failure (AKI patients with ICA-AKI 1 received 0.9% sodium chloride solution at a dose of 40 to 60 mL/kg per day or 20% human albumin at a dose of 0.5 g/kg/day for 48 h when they exhibited serum creatinine levels greater than 1.5 mg/dL; those with ICA-AKI 2 and 3 received 20% human albumin at a dose of 1 g/kg/day also for 48 h or packed red blood cells if the haemoglobin level was below 7 g/dL or in cases of gastrointestinal bleeding); [3] in the presence of infection, antimicrobial treatment was administered; and [4] at diagnosis of hepatorenal syndrome type 1, in addition to human albumin (20-30 g/day), patients received terlipressin or noradrenaline.

Peripheral blood was collected at the time of diagnosis of AKI to measure HMGB1 and IL-6 levels. The two biomarkers were measured by the ELISA method (HMGB1 ELISA ST51011, IBL International GmbH, Germany, and Human IL-6 Duoset ELISA, R&D Systems, USA). Serum samples were stored in a freezer at -80°C.

During the hospitalisation period, demographic, clinical, and laboratory data were collected for the investigation of predictive factors of mortality. Demographic variables included age and sex; the clinical variables included Child-Pugh, Model for End-stage Liver Disease (MELD) and MELD-Na scores, ICA-AKI stage, response to treatment with plasma expanders, and the presence of ascites and hepatic encephalopathy; the laboratory variables included creatinine, albumin, total bilirubin, INR, C-reactive protein, and sodium, in addition to the measurement of HMGB1 and IL-6 levels. All data, except the response to treatment of plasma expanders, were obtained at the time of AKI diagnosis.

The outcomes were 30-day and three-month mortality.

The study was approved by the Research Ethics Committee of the Federal University of Minas Gerais, and the patients who agreed to participate in the study signed an informed consent form.

### 2.1. Sample and Statistical Analysis

SPSS software for Windows version 17.0 (SPSS Inc., Chicago, IL) was used to perform the statistical analyses. The normality of the data was assessed using the Shapiro-Wilk test. Categorical variables are expressed as percentages. The associations between variables were analysed using Student's *t*-test, the Mann-Whitney test (according to the data distribution), or the chi-squared test (or Fisher's exact test when appropriate). Variables with a value of *p* < 0.2 were included in the Cox regression analyses. The fit of the Cox regression model was assessed by the deviance test. The serum levels of HMGB1 and IL-6 that best represented accuracy in predicting mortality were obtained using the ROC curve. A *p* value < 0.05 was adopted.

## 3. Results

**Figure 1:**
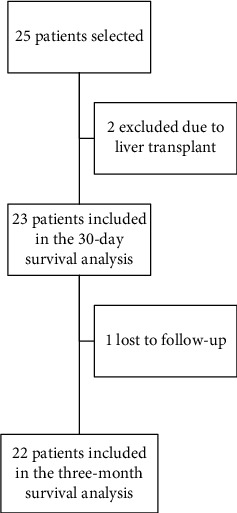
Sample, exclusions, losses, and final number of participants.

Twenty-five patients were selected. Two were excluded from the survival analysis because they underwent liver transplantation during the follow-up period. Thus, 23 patients participated in the 30-day survival analysis. One patient was lost to follow-up and was not included in the three-month survival analysis (Figure 1). The mean age was 55.7 (±9.9) years, and 70% were male. The clinical and laboratory variables of the patients are listed in Table 1.

Of the 23 patients, 15 (65%), seven (30%), and one (4%) were classified as ICA-AKI stages 1, 2, and 3, respectively. Among the causes of AKI, infections accounted for 13 (56%) cases, hypovolemia accounted for nine (39.9%) cases, and renal disease accounted for one (4%) case. Spontaneous bacterial peritonitis was the most common cause of infection (six patients -46%); urinary tract infection, pneumonia, and bacteraemia were present in two cases each (15%); sepsis of uncertain origin in one case (7%). Hypovolemia was most commonly associated with the use of diuretics (six patients -66%), gastrointestinal bleeding (two patients -22%), and diarrhoea (one patient -11%). There was no correlation between causes and AKI stages.

Clinical and laboratory variables were compared between survivors and nonsurvivors at 30 days and three months (Tables 2 and 3). Patients who were alive at the end of the first month had lower Child-Pugh, MELD and MELD-Na scores, INRs, and HMGB1 levels than did the nonsurvivors (10.3 versus 12.7, *p* = 0.019; 20.2 versus 31.5, *p* = 0.05; 23.1 versus 27.6, *p* = 0.015; 1.5 versus 3.1, *p* = 0.009; and 1174.2 pg/mL versus 3338.5 pg/mL, *p* = 0.035, respectively). Regarding serum IL-6 levels, the values were 43.3 pg/mL versus 153.3 pg/mL, respectively (*p* = 0.061). Patients who responded to treatment were more common among survivors (*p* = 0.005). At three months, the MELD-Na score was significantly lower in the group of patients who survived (22.3 versus 29.4, *p* = 0.044). In addition, the ICA-AKI stage also differed between the survivor and nonsurvivor groups (*p* = 0.045). At this time point, HMGB1 levels did not differ between survivors and nonsurvivors (1540 pg/mL versus 2352 pg/mL, *p* = 0.243). The serum IL-6 levels were 35.8 pg/mL versus 87.9 pg/mL (*p* = 0.071). The mortality rate in this sample was 17.4% at 30 days and 30.4% at three months. The most common causes of death were hepatic failure (four cases), followed by acute intestinal bleeding (two cases) and respiratory failure and septic shock (one case each).

A Cox regression model was used to study the various predictors of mortality. Analysis of the 30-day outcome showed that the following variables had a *p* value less than 0.2: Child-Pugh, MELD and MELD-Na scores, response to treatment with plasma expanders, total bilirubin, INR, sodium, IL-6, and HMGB1. For the 90-day outcome, the variables initially included in the Cox model were Child-Pugh, MELD and MELD-Na scores, response to treatment with plasma expanders, ICA-AKI stage, creatinine, and IL-6. After statistical analysis, the model did not identify any variables with a value of *p* < 0.05 that were related to 30-day mortality. In the analysis of prediction of three-month mortality, the only variable that remained associated with mortality was response to treatment with plasma expanders (hazard ratio 5.7, CI 0.27-0.41, *p* = 0.012).

**Figure 2:**
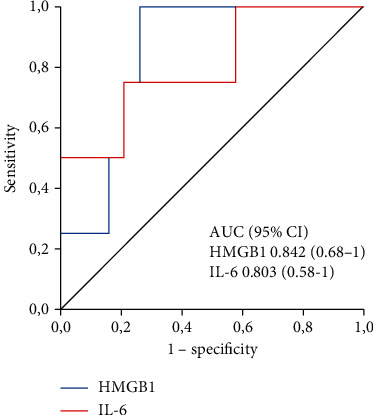
Receiver operator characteristic (ROC) curve and area under the curve (AUC) of HMGB1 and IL-6 in predicting one-month mortality. AUC HMGB1 = 0.842 (0.68–1); AUC IL‐6 = 0.803 (0.58–1).

**Figure 3:**
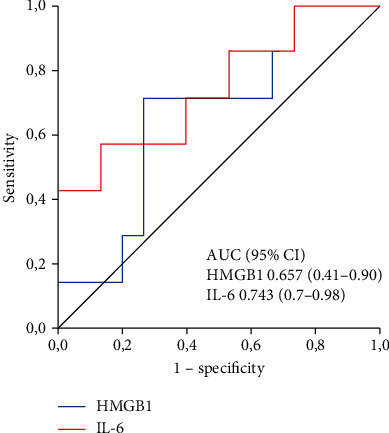
Receiver operator characteristic (ROC) curve and area under the curve (AUC) of HMGB1 and IL-6 in predicting three-month mortality. AUC HMGB1 = 0.657 (0.41–0.9); AUC IL‐6 = 0.743 (0.7–0.98).

Once the ROC curves were obtained, the cut-off values of the serum cytokine levels that most accurately predicted 30-day mortality were 2.168 pg/mL and 73 pg/mL for HMGB1 and IL-6, respectively. These values were associated with sensitivity and specificity rates of 100% and 74%, respectively, for serum HMGB1 levels, and 75% and 79%, respectively, for serum IL-6 levels. The area under the curve was 0.842 for HMGB1 and 0.803 for IL-6 (Figure 2). In the analysis of three-month mortality, the cut-off value of HMGB1 was the same, and that of IL-6 was 45 pg/mL. The sensitivity and specificity were 71% and 73% for HMGB1 and 71% and 60% for IL-6, with areas under the curves of 0.657 and 0.743, respectively (Figure 3).

## 4. Discussion

AKI is often related to circulatory dysfunction in cirrhosis, a condition for which the main risk trigger is splanchnic arterial vasodilation due to portal hypertension [16]. Neurohormones and other substances, such as vasopressin, angiotensin, aldosterone, epinephrine, and norepinephrine, are produced to compensate for the initial depletion of effective intravascular volume. Therefore, an attempt is made to restore intravascular volume through ionic and fluid retention and release of molecules that would restore peripheral vascular resistance and cardiac output, which characterises the hyperdynamic circulation of cirrhotic patients [17].

Nevertheless, hyperdynamic circulation is not the only pathophysiological component of kidney dysfunction in cirrhosis. In fact, acute kidney failure, as well as other forms of organ dysfunction in cirrhosis, can occur in the absence of circulatory decompensation because it is secondary from the complicated interplay between the innate immune system and bacterial products—also known as pathogen-associated molecular patterns (PAMPs)—and antigens from dying cells, called damage-associated molecular patterns (DAMPs) [18–20]. Once connected to pattern recognition receptors, the PAMPs and DAMPs trigger various proinflammatory responses that, when excessive or chronic, can cause tissue damage [6, 18]. Therefore, AKI in cirrhosis results not only from arterial vasodilation but also from uncontrolled systemic inflammation [16]. In fact, kidney failure is one of the main components of ACLF, which is a syndrome that, unlike “simple” acute decompensation of liver cirrhosis, is characterised by marked systemic inflammation, organ failure, and high short-term mortality [4, 5]. The mediators of acute or chronic inflammatory states that increase systemic vasodilation and are associated with AKI include HMGB1, a type of DAMP, and IL-6 [21].

In this study, we showed that HMGB1 levels could differentiate survivors from nonsurvivors at 30 days (median of 1174 pg/mL versus 3338 pg/mL, *p* = 0.035). Regarding IL-6, the *p* value does not allow concluding that the median values of the two groups differed from each other (43.3 pg/mL versus 153.3 ng/mL, *p* = 0.061). In the performance analysis, the two cytokines showed satisfactory discrimination of survivors from nonsurvivors at 30 days (areas under the ROC curve of 0.842 and 0.803 for HMGB1 and IL-6, respectively). At three months, unlike the first time point, the median serum levels of HMGB1 and IL-6 were not lower in the survivors (1540 ng/mL versus 2352 ng/mL, *p* = 0.243 and 35.8 ng/mL versus 87.9 ng/mL, *p* = 0.071, respectively). However, the behaviour of IL-6 at three months was quite similar to that at 30 days, as there was a tendency towards significance (*p* = 0.061 at 30 days and *p* = 0.071 at three months). At three months, the area under the ROC curve of the two cytokines was also smaller. The areas under the curves were 0.657 and 0.743, which were associated with sensitivity and specificity rates of 71% and 73% for HMGB1 and of 71% and 60% for IL-6. These results suggest that both cytokines may be good predictors of mortality, especially in the short term.

It is possible to state that the association between HMGB1—and possibly IL-6—and survival is not an epiphenomenon because both cytokines are involved in the pathophysiology of AKI-associated organ failure [21–23]. In experimental models in which AKI was induced by bilateral nephrectomy or ischaemia, for example, it was possible to identify liver tissue damage, characterised by increases in leukocyte and cytokine influx, oxidative stress, and apoptosis [24–27]. However, the interaction between the liver and the kidneys is bidirectional; AKI in cirrhotic patients seems to be secondary not only to circulatory failure but also to inflammatory reactions mediated by TLR-4, among other mechanisms [28]. In fact, increased TLR-4 receptor levels were found in renal tubular cells of cirrhotic patients with AKI compared to those without AKI [29]. In animal models of induced cirrhosis, intestinal decontamination with norfloxacin, after the injection of bacterial lipopolysaccharides, was able to reduce the renal expression of these receptors, suggesting that they may mediate kidney injury in the context of infection or systemic inflammation [30]. In addition, blockage of HMGB1 by monoclonal antibodies protects against AKI and ACLF [31, 32]. Although it is possible that the two cytokines may increase with liver damage, both HMGB1 and IL-6 showed no significant correlation with Child-Pugh and MELD scores in our cohort.

Since serum HMGB1 and IL-6 can accumulate in urine during AKI-associated inflammatory processes [13, 33], both could be useful in the differential diagnosis of the causes of AKI in cirrhosis patients—especially between parenchymal renal disease and functional AKI—similar to other urinary biomarkers [34]. However, since the absence of response to volume expansion is an important predictor of a poor prognosis in cirrhotic patients with AKI irrespective of its aetiology, the biomarkers' ability to differentiate between nonresponders and responders could reduce the costs and adverse effects of unnecessary treatments as well as optimise the allocation of patients on liver transplant waiting lists. In our study, HMGB1 and IL-6 showed good performance in predicting the absence of response to treatment (area under the ROC curve of 0.833 and 0.881, respectively), with a sensitivity of 100% and specificities of 67.7% and 76.8%%, respectively, for cut-off values of 73.6 pg/mL of IL-6 and 2168 pg/mL of HMGB1. In the multivariate analysis, the only variable that was associated with three-month mortality was the response to treatment with plasma expanders.

## 5. Conclusions

A literature search showed no other study that evaluated the role of HMGB1 and IL-6 as predictors of mortality in cirrhotic patients with AKI. In our cohort, HMGB1 levels were associated with decreased survival in cirrhotics; both biomarkers showed good performance in predicting short-term mortality. However, our study has limitations, such as the small size of our sample and its observational design. New studies with a larger number of patients are needed to evaluate the role of these cytokines in organ failure in cirrhosis and in the prognosis of cirrhotic patients with AKI.

## Figures and Tables

**Table 1:** Clinical and laboratory variables of 23 cirrhotic patients with AKI.

Characteristics (*n* = 23)	Mean/median/*n* (%)
Age	55.7
Gender (male/female)	16 (70%)/7 (30%)
Mean blood pressure (mmHg)	64 (±5.8)
Body weight (kg)	72 (±7.7)
Child-Pugh score	10.8 (±1.9)
Child-Pugh class (A/B/C)	0/9 (39%)/14 (71%)
MELD	22.17 (±7.7)
MELD-Na	24.6 (±7.6)
Use of medications	
Diuretics	20 (87%)
Nephrotoxic agents	1 (4%)
AKI stages	
1	15 (65%)
2	7 (30%)
3	1 (4%)
AKI causes	
Infection	13 (56%)
Hypovolemia	9 (39%)
Hepatorenal syndrome	0
Renal disease	1 (4%)
HMGB1 (pg/mL)	1697.2 (0.1-6851.8)
IL-6 (pg/mL)	47.6 (0.1-1107.3)
Creatinine (mg/dL)	1.99 (±0.7)
Total bilirubin (mg/dL)	4.6 (0.4-15.3)
Albumin (g/dL)	2.5 (±0.4)
INR	1.6 (1.0-5.0)
Sodium (mEq/L)	133.6 (±4.8)
C-reactive protein (mg/L)	34 (9-287)

AKI: acute kidney injury.

**Table 2:** Comparison of demographic, clinical, and laboratory variables between survivors and nonsurvivors at 30 days.

	Survivors (*n* = 19)	Nonsurvivors (*n* = 4)	*p* value
Demographic			
Age	56.1 (±10.4)	54 (±7.7)	0.709
Males	14 (73.7%)	2 (50%)	0.349
Females	5 (26.3%)	2 (50%)	
Clinical			
Child-Pugh	10.3 (±1.7)	12.7 (±1.5)	0.019
MELD	20.2 (±6.1)	31.5 (±8.6)	0.05
MELD-Na	23.1 (±6.4)	27.6 (±7.3)	0.015
ICA-AKI stage			
1	13 (68.4%)	2 (50%)	0.609
2	5 (26.3%)	2 (50%)	
3	1 (5.3%)	0	
AKI causes			
Infection	11 (57.9%)	2 (50%)	0.822
Hypovolemia	7 (36.8%)	2 (50%)	
Hepatorenal syndrome	0	0	
Renal disease	1 (5.3%)	0	
Response to treatment with plasma expanders			
Complete	12 (63.2%)	1 (25%)	0.005
Partial	7 (36.8%)	1 (25%)	
Absent	0	2 (50%)	
Encephalopathy			
Present	6 (31.6%)	2 (50%)	0.589
Absent	13 (68.4%)	2 (50%)	
Ascites			
Present	17 (89.5%)	4 (100%)	0.497
Absent	2 (17.1%)	0	
Laboratory			
HMGB1 (pg/mL)	1174.2 (0.1-6665)	3338.5 (2273-6851.8)	0.035
IL-6 (pg/mL)	43.3 (0.1-135.6)	153.3 (21.1-1107)	0.061
Creatinine (mg/dL)	2.0 (±0.7)	1.8 (±0.1)	0.663
Total bilirubin (mg/dL)	1.7 (0.4-15.3)	6 (4.6-8.0)	0.168
Albumin (g/dL)	2.5 (±0.4)	2.4 (±0.6)	0.481
INR	1.5 (1.0-2.4)	3.1 (2.0-3.09)	0.009
Sodium (mEq/L)	133.2 (±5.4)	134.8 (±4.3)	0.127
C-reactive protein (mEq/L)	34 (9-287)	28 (18-46)	0.626

ICA-AKI: International Club of Ascites-Acute Kidney Injury.

**Table 3:** Comparison of demographic, clinical, and laboratory variables between survivors and nonsurvivors at three months.

	Survivors (*n* = 15)	Nonsurvivors (*n* = 7)	*p* value
Demographic			
Age	55.3 (±11.6)	53.9 (±5.7)	0.528
Males	17 (65.4%)	16 (72.7%)	0.584
Females	9 (34.6%)	6 (33.3%)	
Clinical			
Child-Pugh	10.3 (±1.7)	11.6 (±2.1)	0.134
MELD	20.1 (±6.1)	26.9 (±9.8)	0.061
MELD-Na	22.3 (±7.0)	29.4 (±7.5)	0.044
ICA-AKI stage			
1	12 (80%)	2 (28.6%)	0.045
2	3 (20%)	4 (57.1%)	
3	0 (11.5%)	1 (14.3%)	
AKI causes			
Infection	8 (53.3%)	4 (57.1%)	0.783
Hypovolemia	6 (40%)	3 (42.9%)	
Hepatorenal syndrome	0	0	
Renal disease	1 (6.7%)	0	
Response to treatment with plasma expanders			
Complete	10 (66.7%)	2 (28.6%)	0.059
Partial	5 (33.3%)	3 (42.9%)	
Absent	0	2 (28.6%)	
Encephalopathy			
Present	4 (26.7%)	3 (42.9%)	0.387
Absent	11 (73.3%)	4 (57.1%)	
Ascites			
Present	13 (86.7%)	7 (100%)	0.455
Absent	2 (13.3%)	0 (9.1%)	
Laboratory			
HMGB1 (pg/mL)	1540 (0.1-6665)	2352 (53.2-6851.8)	0.243
IL-6 (pg/mL)	35.8 (0.1-113.5)	87.9 (4.4-1107.3)	0.071
Creatinine (mg/dL)	1.9 (±0.6)	2.4 (±0.8)	0.086
Total bilirubin (mg/dL)	1.7 (0.4-15.3)	5.37 (0.4-8.3)	0.573
Albumin (g/dL)	2.5 (±0.4)	2.6 (±0.6)	0.525
INR	1.5 (1-2.24)	2.0 (1.2-5.0)	0.341
Sodium (mEq/L)	133.3 (±4.4)	132.8 (±5.5)	0.829
C-reactive protein (mEq/L)	30.8 (5-278)	31.6 (5-287)	0.860

ICA-AKI: International Club of Ascites-Acute Kidney Injury.

## Data Availability

Answer: Yes. Comment: The study data is available with the corresponding author upon request.
